# A survey on pulmonary screening practices among otolaryngology-head & neck surgeons across Canada in the post treatment surveillance of head and neck squamous cell carcinoma

**DOI:** 10.1186/s40463-015-0057-7

**Published:** 2015-02-04

**Authors:** J Madana, Gregoire B Morand, Luz Barona-Lleo, Martin J Black, Alex M Mlynarek, Michael P Hier

**Affiliations:** Department of Otolaryngology-Head and Neck Surgery, Sir Mortimer B. Davis-Jewish General Hospital, McGill University, 3755 Côte Ste-Catherine Road, Montreal, QC Canada; Department of Otolaryngology-Head and Neck Surgery, Wayne State University, Detroit, MI USA

**Keywords:** Head and neck cancer, Pulmonary screening, Survey, Canadian head and neck surgeons

## Abstract

**Background:**

Post treatment lung screening for head and neck cancer patients primarily focuses on the distant metastasis and a high rate of second primary can also be expected. The best screening tool and timing for this purpose is controversial. We sought out to assess the current practice and beliefs among Canadian Head and Neck Surgeons.

**Methods:**

After Ethical Board approval, a nationwide survey was conducted through the Canadian Society of Otolaryngology (CSO) among head and neck surgeons regarding their practices for pulmonary screening in HNSCC patients.

**Results:**

Our CSO survey among Otolaryngology-head and neck surgeons showed that 26 out of 32 respondents perform routine lung screen, out of which 23 (88%) feel that chest radiography should be preferred. The majority of respondents felt that lung screening could impact beneficially on mortality. For symptomatic patients, low-dose spiral CT was the preferred modality (48%), followed by PET/CT scan (14%) and sputum cytology (14%). In high-risk asymptomatic patients (current smoker, radiation exposure, family history and advanced HNSCC), 31% of respondents performed a CXR. The same percentage performed a low dose CT, while 19% relied on PET scan. A further 19% of respondents did not perform any screening in high-risk patients. Most respondents (77%) had more than 10 years practice since graduation from medical school and came from the provinces of Quebec, Ontario and Alberta.

**Conclusion:**

Chest radiography remains the preferred modality for lung screening and was believed to be impacting beneficially on lung mortality. The recent literature does not seem to be in agreement with those beliefs. Further studies to establish which modality is best and concurrent nation-wide education are warranted.

## Introduction

In head and neck squamous cell carcinoma patients (HNSCC), post treatment surveillance for distant disease is mostly focusing on the lungs, as HNSCC distant metastasis occurs in this organ in 90% of the cases and a high rate of primary of the lungs can be expected due to field cancerization of the entire upper aerodigestive tract [1-4]. Furthermore distant metastasis isolated to other sites such as liver or bones are rare in the absence of simultaneous pulmonary malignancy [4]. The traditional method for lung screening has been chest radiography, as it is widely available, cheap and has a low radiation dose, which allows for safe repetitive screening [5,6].

Screening for distant metastasis and/or second primary of the lungs is useful as it allows for prognostication and adapted patient counselling [7] and in case of early detection may impact beneficially the prognosis of patients [8]. Early stage lung primary can be treated with curative intent [9] and some reports have claimed some benefit of metastasectomy in selected patients [8]. Overall, screening for distant metastasis has gained importance in the last few years, as aggressive locoregional treatment in HNSCC has led to better locoregional control but higher risk of distant failure [10]. This reverse failure pattern was first reported by Vikram *et al.* [10], who showed that only 15% of patients relapsed above the clavicle, as opposed to 70% in historical series.

Chest radiography is, however, likely to be out-dated, as previously shown by several groups [5,11]. Newer technologies now widely available such as computer tomography and positron emission tomography (PET/CT) are currently being evaluated as screening tools in HNSCC [4,5,12-14].

The objective of this study was to evaluate the current beliefs and practice among Head and Neck surgeon members of the Canadian Society of Otolaryngology using a nation-wide survey. The results of this survey were compared to latest data from the literature in the discussion.

## Methods

After Ethics Review Board approval, a nationwide survey was conducted through the Canadian Society of Otolaryngology (CSO) among Otolaryngology-head and neck surgeons regarding their practices for post treatment pulmonary screening in HNSCC. A simple survey with 6 questions regarding actual practice was designed previously, reviewed, and sent to head and neck surgeons across Canada. A sample of the survey is available in Table 1.

**Table 1:** **Structure of original questionnaire for nation-wide survey**

**Questions**	**Possible responses**	
1. How do you perform routine lung screening during the post treatment follow-up of head and neck cancer	Lung radiography	All patients
	Low-dose CT	Only symptomatic patients
	PET/CT	Only high risk patients (smokers, radiation exposure, family history and advanced HNSCC)
	Sputum cytology	
	Physical exam	
	No routine screen	
2. What is the frequency and duration of lung screening in head and neck cancer during follow up in your practice	5 years	Biennially
	10 years	Annually
	Lifelong	Half-yearly
3. How effective do you believe the screening procedures listed in question 1 are in reducing lung cancer mortality during the follow-up of head and neck cancer	Very effective	
	Somewhat effective	
	No effective	
	Don’t know	
4. Have any of your patients during the past 12 months inquired about lung screening	Yes	
	No	
5. Number of years of your clinical head and neck practice and years since graduation from medical school	0-5 years	
	6-10 years	
	11-20 years	
	More than 20 years	
6. What is your practicing census region and the patient volume during a typical week of your head & neck practice	Alberta	<75 patients/week
	Manitoba	
	Saskatchewan	
	British Columbia	
	New Brunswick	75-125 patients/week
	Nova Scotia	
	Prince Edward Island	
	Newfoundland and Labrador	
	Northwest Territories	>125 patients/week
	Nunavut	
	Ontario	
	Quebec	
	Yukon	

## Results

**Figure 1:**
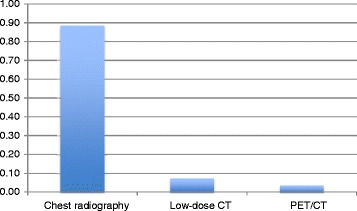
**Preferred modality in asymptomatic patients among COS head and neck surgeons.**

Our CSO survey among Otolaryngologist - Head and Neck surgeons showed that 26 out 32 (81%) respondents perform routine lung screening, out of which 23 (88%) feel that chest radiography should be preferred versus low dose CT scan or PET scan for annual pulmonary screening among all asymptomatic HNSCC patients during follow up (Figure 1). In asymptomatic patients, only 2 respondents did not perform any lung screening. The response rate of the survey was 84.2% (32 out of 38) and the survey was sent only once.

For symptomatic patients, low-dose spiral CT was the preferred modality (48%), followed by PET/CT scan (14%) and sputum cytology (14%).

In high-risk asymptomatic patients (current smoker, radiation exposure, family history and advanced HNSCC), 31% of respondents performed a CXR. The same percentage performed a low dose CT, while 19% relied on PET scan. A further 19% of respondents did not perform any screening in high-risk patients.

Seventeen of 28 respondents (60%) request lung screening for 5 years, while 4 (15%) and 7 (25%) do it for 10 years and life long, respectively. Most respondents were screening their patients annually (74%), while less than 15% did it biennially or half-yearly, respectively.

CXR was believed to be in 11% “very effective” and 50% “somewhat effective” in reducing lung malignancy mortality during HNSCC follow-up for non-smoker. In former smokers, the believed efficacy was 14% and 57%, respectively. In current smoker, CXR was believed to be “very effective” and “somewhat effective” in 27% and 54%, respectively. Seventy-four percent of respondents did not recall having any patient inquiring about lung screening in the past 12 months. Most respondents (77%) had more than 10 years practice since graduation from medical school and came from the provinces of Quebec, Ontario and Alberta (11, 10, and 6, respectively).

## Discussion

The main goal of this study was to assess the practice among head and neck surgeons in Canada for post treatment screening. Briefly, we showed that the preferred modality was chest radiography, done yearly in all patients for 5 years and this was believed in the majority of cases to be effective. Let us discuss each part of this statement point by point, in light of the classic Wilson and Jungner screening criteria (Table 2) [15].

**Table 2:** **Wilson and Jungner screening criteria**

1.	The condition sought should be an important health problem.
2.	There should be an accepted treatment for patients with recognized disease.
3.	Facilities for diagnosis and treatment should be available
4.	There should be a recognizable latent or early symptomatic stage.
5.	There should be a suitable test or examination.
6.	The test should be acceptable to the population.
7.	The natural history of the condition, including development from latent to declared disease, should be adequately understood.
8.	There should be an agreed policy on whom to treat as patients.
9.	The cost of case finding (including diagnosis and treatment of patients diagnosed) should be economically balanced in relation to possible expenditure on medical care as a whole.
10.	Case finding should be a continuing process and not a “once and for all” project.

### Target group

As screening is less efficacious if the expected prevalence is low according to the Bayes theorem, screening has to target particular groups in order to be cost effective and avoid high rate of false positive [16]. Previous studies have shown that patients with advanced tumor stage, tumor from the hypopharynx and smokers are at higher risk for pulmonary malignancy [1,17-19]. In human papilloma virus patients, the rate of pulmonary malignancy may be lower due to lack of field carcinogenesis and a somewhat different distant metastatic pattern [20,21], thus requiring different strategies not only in treatment but also in surveillance.

### Screening accuracy and impact on mortality

Although chest radiography remains the preferred and most widely used screening modality, numerous studies showed that regular chest radiography was not efficacious at detecting pulmonary malignancy [5,6,11]. More sensitive methods are required. Computer chest tomography (CT), positron emission tomography (PET/CT) and whole body MRI (WBMRI) are actually being evaluated [4,5,12,13,22,23]. CT is now widely available, has a reported sensitivity and specificity of 73% and 86%, respectively [22] and recent development such as low dose CT may compensate for the relative high radiation dose of each examination, a factor particularly important for repetitive imaging such as in yearly screening [24]. For lung cancer, it was shown that LDCT could reduce mortality in some high-risk group such as smokers when LDCT was used as a screening tool [25]. A recent Cochrane systematic review came to the conclusion that LDCT could be beneficial in a high-risk lung cancer group, but not chest radiography or sputum cytology [26]. In HNSCC, to our knowledge, no trials are available yet.

PET/CT is deemed to detect more metastasis and earlier in their course [12,13]. This advantage however could not be translated into clinical benefit for the patients in any studies [4,13]. It must be noted that most of the studies were retrospective and that the diagnosis of distant metastasis mostly did not change the treatment, as classically only symptomatic patients will receive palliative chemotherapy but no specific intervention was performed in early diagnosis patients [27]. Classically patients with distant metastasis are treated only when symptomatic, leading some authors to argue that screening may have low clinical impact, and is unlikely to change treatment course [27]. This may be true for distant metastasis, however second lung primary may be potentially treated with curative intent and thus remains important. Furthermore, when interpreting the literature, one should be cautious not to interpret the prolonged time between diagnosis and death as survival advantage, as this may be explained by lead-time bias [6].

Although PET/CT screens the whole body and this may seem like a critical advantage, a recent study by Spector *et al.* [4] showed that the most common site for asymptomatic metastasis were the lungs, whereas patients who developed bone, liver or brain metastasis were typically identified by imaging initiated by symptoms. This would suggest that screening (per definition in asymptomatic patients) may be sufficient if performed in the lungs only, as metastasis to other organs are likely to become rapidly symptomatic and not “detectable” in early disease course [4]. However, in the province of Ontario, PET/CT is not approved for routine pulmonary screening and therefore would not be an option for any surgeon responding from Ontario. PET/CT scan was believed to be useful for screening by 19% of respondents (2 from Quebec; and one from Saskatchewan, Alberta, British Columbia and New Brunswick each). Baring the Ontario respondents (10 in number, 31% of total), 27% (6/22) of the respondents from provinces other than Ontario think PET/CT scan is a viable screening tool in high risk head and neck cancer patients on follow up. Finally newer studies have shown that WBMRI can be used as screening tool for HNSCC patients. WBMRI was shown in a pilot study to be accurate and not to require very long imaging times [23]. Further studies on that modality are required.

For sputum cytology, the scarce evidence available on that matter failed to support any potential use in HNSCC patients or any lung cancer case [26,28].

### Timing and frequency of screening

To our knowledge, the adequate timing and frequency of screening for lung metastasis is based on empirical evidence. Most head and neck surgeons in Canada preferred yearly screening for 5 years. As distant metastasis most commonly occurs within 2 years after the diagnosis [29], one could wonder if half yearly screening in the first 2 years followed by yearly screening afterwards would be more adequate [30]. Again, although this may detect distant disease earlier in the course, it remains to be proven that this impacts survival beneficially and does not have negative impact on the health care system [31]. For screening tools with poor sensitivity such as chest radiography, a recent study showed it was ineffective when performed twice yearly [31].

## Conclusion

Chest radiography remains the preferred screening modality for distant metastatic HNSCC in Canada, despite lacking sensitivity. Hence the lung screening practices of head and neck surgeons in Canada are contrary to what the evidence would suggest, and cannot be justified.
